# Intestinal Microbiome Shifts, Dysbiosis, Inflammation, and Non-alcoholic Fatty Liver Disease

**DOI:** 10.3389/fmicb.2018.00061

**Published:** 2018-01-30

**Authors:** Emma T. Saltzman, Talia Palacios, Michael Thomsen, Luis Vitetta

**Affiliations:** ^1^Sydney Medical School, University of Sydney, Sydney, NSW, Australia; ^2^Medlab Clinical, Sydney, NSW, Australia

**Keywords:** intestinal microbiome, intestinal epithelial cell dysbiosis, dysbiosis, macrophage, inflammation, mucosal immunity, NAFLD

## Abstract

Adverse fluctuations in the distribution of the intestinal microbiome cohort has been associated with the onset of intra- and extra-intestinal inflammatory conditions, like the metabolic syndrome (MetS) and it's hepatic manifestation, non-alcoholic fatty liver disease (NAFLD). The intestinal microbial community of obese compared to lean subjects has been shown to undergo configurational shifts in various genera, including but not limited to increased abundances of *Prevotella, Escherichia, Peptoniphilus*, and *Parabacteroides* and decreased levels of *Bifidobacteria, Roseburia*, and *Eubacteria* genera. At the phylum level, decreased *Bacteroidetes* and increased *Firmicutes* have been reported. The intestinal microbiota therefore presents an important target for designing novel therapeutic modalities that target extra-intestinal inflammatory disorders, such as NAFLD. This review hypothesizes that disruption of the intestinal–mucosal macrophage interface is a key factor in intestinal-liver axis disturbances. Intestinal immune responses implicated in the manifestation, maintenance and progression of NAFLD provide insights into the dialogue between the intestinal microbiome, the epithelia and mucosal immunity. The pro-inflammatory activity and immune imbalances implicated in NAFLD pathophysiology are reported to stem from dysbiosis of the intestinal epithelia which can serve as a source of hepatoxic effects. We posit that the hepatotoxic consequences of intestinal dysbiosis are compounded through intestinal microbiota-mediated inflammation of the local mucosa that encourages mucosal immune dysfunction, thus contributing important plausible insight in NAFLD pathogenesis. The administration of probiotics and prebiotics as a cure-all remedy for all chronic diseases is not advocated, instead, the incorporation of evidence based probiotic/prebiotic formulations as adjunctive modalities may enhance lifestyle modification management strategies for the amelioration of NAFLD.

## Introduction

NAFLD is a growing public health concern, laying claim to both a steadily rising prevalence as well as an increasingly young age at diagnosis (Welsh et al., 2013; Nobili et al., 2014). These trends reflect the increased rates of risk factors associated with an obesogenic lifestyle and the development of type 2 diabetes mellitus (T2DM) (Targher et al., 2007; Welsh et al., 2013). The prevalence of NAFLD in obese adults or those with T2DM has been reported to be 67.5 and 74%, respectively, (Williams et al., 2011; Paquissi, 2016) compared to only 25% in the general adult population (Younossi et al., 2016). As a spectrum of diseases, NAFLD has been associated with significant morbidity and mortality, with advanced forms of the disease expressed as fibrosis, cirrhosis, and hepatocellular carcinoma (HCC).Additionally, insulin resistance (IR) and obesity have been identified as NAFLD risk factors (Gaggini et al., 2013), with NAFLD also reported to increase the risk of T2DM and cardiovascular disease, justly classifying NAFLD as the hepatic manifestation of the metabolic syndrome (MetS) (Adams et al., 2005; Targher et al., 2007, 2010; Dunn et al., 2008; Starley et al., 2010; Gregor and Hotamisligil, 2011; Lumeng and Saltiel, 2011; Dietrich and Hellerbrand, 2014; Paolella et al., 2014; Paquissi, 2016). NAFLD hence presents as a multi-systemic disease (Starley et al., 2010; Paolella et al., 2014). In light of the potential of NAFLD to progress to an ever-increasing prevalence, an appreciation of the molecular mechanisms that facilitate the manifestation of NAFLD disease chronicity and the pathways that trigger the transition across the spectrum of diseases is a pertinent aspect in designing novel effective therapeutic modalities. With the purported pathogenic mechanisms of NAFLD being intertwined with peripheral IR, increased liver lipolysis is reported to contribute to increased levels of hepatic free fatty acids (FFAs) (Paolella et al., 2014). IR simultaneously triggers increased gluconeogenesis and reduced glyconeogenesis, which further increases the production of circulating FFAs (Paolella et al., 2014).

The definition of NAFLD covers a spectrum of liver histologies, ranging from the benign and usually non-progressive simple steatosis (SS) (Tilg and Moschen, 2010; characterized by the accumulation of lipid droplets that exceed 5% of the total weight of the liver) to non-alcoholic steatohepatitis (NASH) which is found in 30% of NAFLD patients and is described as the beginning stages of inflammation and lobular ballooning which results from persistent hepatic injury (Jiang et al., 2015). Chronic inflammation can cause the liver to respond with compensatory tissue repair, a mechanism that initiates fibrosis and even cirrhosis through collagen deposition and scarring—which eventually forms the foundation of hepatocellular cancer (HCC) in extremely rare cases (Tilg and Moschen, 2010; Bieghs and Trautwein, 2014). Systems biology continues to probe the pathophysiology of this disease and the factors that establish it's manifestation and drives disease progression across the spectrum toward severe phenotypes.

The manifestation and progression of NAFLD is tentatively attributed to a range of factors as part of the multiple *parallel hits* hypothesis, which postulates that NAFLD is the result of inflammation in the liver induced by numerous intestinal-derived or adipose tissue-derived triggers (Tilg and Moschen, 2010). With the *first hit* said to be the onset and maintenance of SS, and the additional hits of gut-derived endotoxins and pro-inflammatory cytokines from adipose tissue reported to provide the impetus for NASH development and subsequent progression (Day and James, 1998). This hypothesis is flagging the importance of aberrant innate immunity as a central pathway for NAFLD progression (Miele et al., 2009; Tilg and Moschen, 2010) along with stress signaling networks and circulating adipocytokines and pro-inflammatory cytokines (Miele et al., 2009; Tilg and Moschen, 2010).

Although the molecular pathways that lead to the pathogenesis and progression of NAFLD remain poorly understood, it is accepted that inflammation is a major factor in hepatic injury (Bieghs and Trautwein, 2014). Currently, experimental data suggests that interactions of the innate immune system with the different resident liver cell types help perpetuate and maintain adverse inflammatory responses in the liver (Starley et al., 2010; Farrell et al., 2012; Bieghs and Trautwein, 2014). Serum markers of inflammation, including C-reactive protein (CRP), interleukins (ILs), and other general immunity markers are associated with the diagnosis and prognosis of NAFLD (Chiang et al., 2010; Harley et al., 2014), whilst at the cellular level, data has implicated an imbalance in T helper 17 (Th17) cells over regulatory T (Treg) cells, that occurs from an over-differentiation of T helper cells (Hammerich et al., 2011). A disturbance to the equilibrium between Th17 and Treg cells is a key event in the initiation of pro-inflammatory activity.

The innate immune system responds to cell damage or pathogenic invasion through pattern recognition receptors (PRRs), that are expressed intracellularly or on the surface of resident liver cells (Bieghs and Trautwein, 2014). These PRRs are programmed to detect damage-associated molecular patterns (DAMPs) that are released by injured cells or pathogen-associated molecular patterns (PAMPs), which are derived from intestinal bacterial metabolites (Pedra et al., 2009; Takeuchi and Akira, 2010). From a consideration of the consequences of intestinal epithelial dysbiosis, this review hypothesizes that the cascade of signals that activate adverse innate immune system and inflammatory activity implicated in NAFLD pathophysiology are triggered by the continuous release of endotoxins and other intestinal bacterial-derived products which can reach the liver through the gut-liver axis interface.

Resident liver cells have their own PRRs in toll-like receptors (TLRs). Activating these TLRs is an important step in the development of NAFLD as they are responsible for inducing gene transcription that facilitates responses of the innate immune system (Takeuchi and Akira, 2010). Kupffer cells, stellate cells, and hepatocytes amongst others, express TLRs and recognize a large array of PAMPs, which enable pro-inflammatory activity by activating different liver cells. PAMPs and DAMPs as well as irritants from the host's environment are themselves recognized by inflammasomes, receptors of the innate immune system. In response to sensing infectious microbes, inflammasomes are responsible for the pro-inflammatory activity observed in the initiation or manifestation of inflammatory diseases, like NAFLD. NLRP3 is an inflammasome that has been reported to be specifically and critically involved in NAFLD progression, with experimental and clinical data identifying higher levels of expression in NASH-affected subjects.

The intestinal microbiome is central to the narrative that NAFLD manifestation is largely a consequence of dysregulated innate immunity in response to persistent pro-inflammatory activity. The role of the intestinal microbiome is multi-factorial, functioning as an immunological, metabolic, and protective tool for optimal host health. When the intestinal microbiome is in dysbiosis, the health of the host is compromised as the microbiome is unable to maintain control of local homeostasis, increasing intestinal permeability. Disruption to intestinal epithelial homeostasis leads to hepatic exposure to exogenous and endogenous antigens that drives hepatoxic influences via the gut-liver axis interface (Glavan et al., 2016).

## The intestinal microbiome and the epithelial barrier

The microbial ecosystem of the gastrointestinal tract (GIT) comprises a metabolically and immunologically complex and active organ (Vitetta et al., 2013). Recognized as the most biodiverse and dense microbial site, the intestinal microbiome is estimated to harbor over 10^14^ bacterial cells (Jiang et al., 2015). Serving as a key influence of host health, the GIT is the site that facilitates the exposure of environmental, dietary, and microbial antigens to the immune system (Vitetta et al., 2013). Existing in a state of symbiotic homeostasis, the intestinal microbiome and the immune system largely co-develop from birth (Nicholson et al., 2012). Subject to an array of complex interactions, dependent on host genetics, lifestyle, dietary and environmental cues, the microbiome and host share a bi-directional relationship, with both parties helping to shape each other's development and composition (Nicholson et al., 2012).

**Figure 1:**
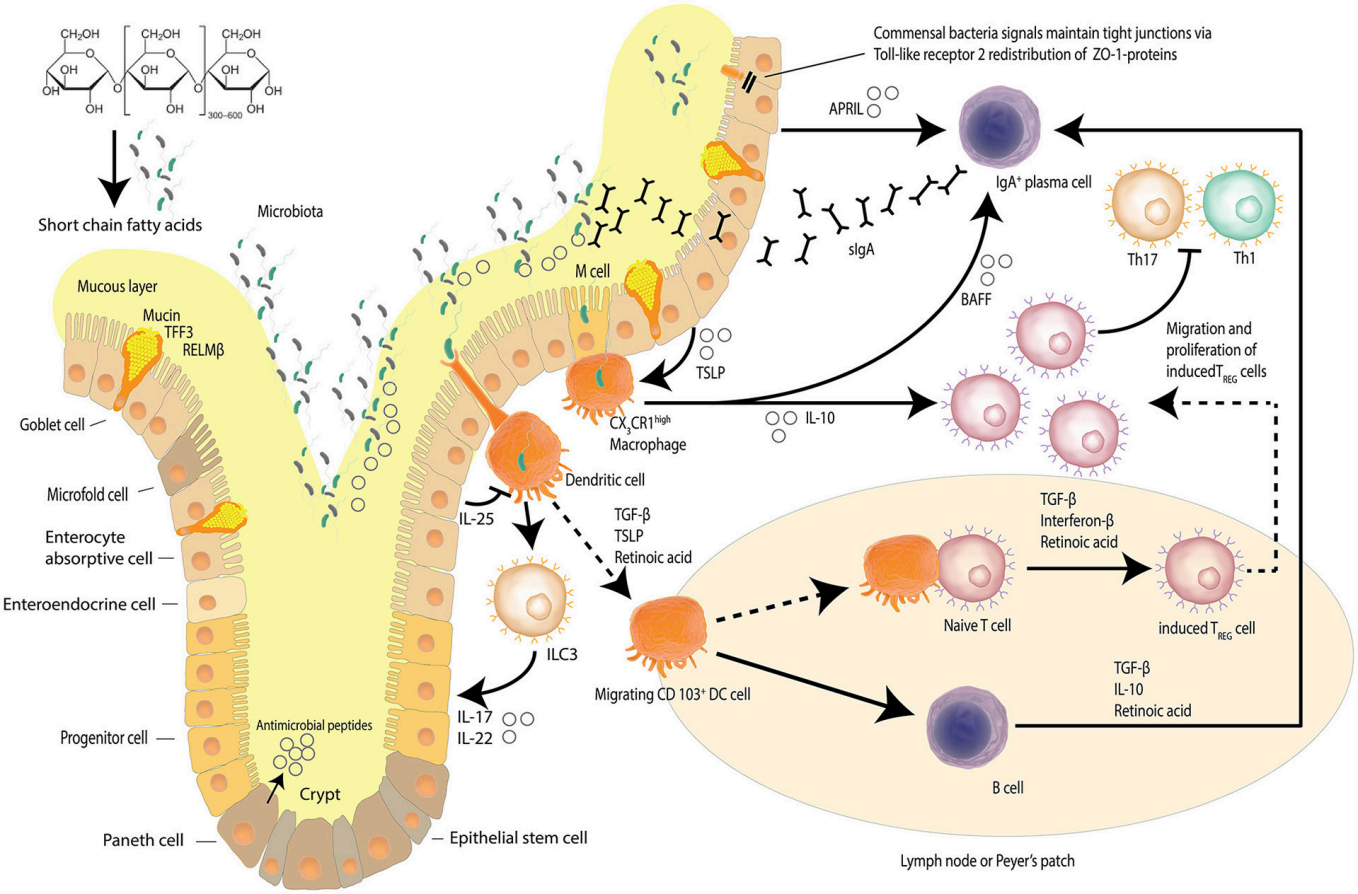
Intestinal epithelial barrier and homeostasis. Several mechanisms, including a mucus layer, antimicrobial peptides and a tight junction protein network collaborate to ensure that the intestinal barrier is not compromised. Goblet cells secrete mucin to provide a protective coating, provide structural integrity and regulate macrophage and adaptive T cell responses during inflammation. Goblet cells also facilitate microbial translocation. The intestinal epithelia network of cells produce a range of soluble protein factors and also express the integrin ligand semaphorin 7A that modulates intestinal CX_3_CR1 macrophage function. Localized CX3CR1 macrophages further release IL-10 to support the proliferation of induced Treg cells and B cell activating factor (BAFF) to further stimulate the production of migrating secretory IgA (sIgA) across the epithelium. Dendritic cells sample bacteria and present antigens to other cells including innate lymphoid cells (ILCs). Dietary starch is converted by intestinal bacteria into short chain fatty acids that serve as a source of energy for the epithelial cells but also act as signaling molecules. Signals from commensal bacteria help maintain tight junction via Toll-like receptor 2 (TLR-2) redistribution of ZO-1 proteins. (Kidd, 2003; Wahl, 2007; Hume, 2008; Ogino et al., 2011; Kayama et al., 2012; Abbas et al., 2013; Smith et al., 2013; Zheng, 2013; Ai et al., 2014; Guilliams et al., 2014; Peterson and Artis, 2014; Gottschalk and Kurts, 2015; Robinson et al., 2015; Nakahashi-Oda et al., 2016) Adapted from Peterson and Artis (2014).

The intestinal epithelial barrier is a dynamic network made up of luminal and mucosal components (Paolella et al., 2014); with an epithelial cell layer interlaced with innate mucosal immunity and neuroendocrine elements, encasing the paracellular space that houses the intestinal microbiome diverse niches (Paolella et al., 2014; Ringel et al., 2015). The single layer of epithelial cells that line the intestines is bound by a tight junction protein (TJP) network, serving to form the physical barrier, which provides protection against potential pathogenic assaults and toxins that increases the risk of systemic sepsis. Intestinal epithelial cells have a rapid time of turnover of between 2 and 6 days (Ramachandran et al., 2000). The TJP network also regulates intestinal permeability, providing the pores and channels for passage of molecules (i.e., water, electrolytes, nutrients) by selective permeability (Paolella et al., 2014). The intestinal barrier is also involved in the co-ordination of responses of the innate immune system, with macrophage/dendritic cell activation contributing to host defenses against microbial-induced systemic infections (Paolella et al., 2014). The protrusions that characterize macrophages/dendritic cells allow for the sensation of potential pathogens that have breached the intestinal mucus layers, the mucosa, as well as those sensed in other parts of the intestinal lumen, resulting in the induction of responses such as phagocytosis and of the acquired immune system through B-cell activation (Kumar et al., 2011; Kinnebrew and Pamer, 2012). Figure 1 details the maintenance of the homeostatic state of the intestinal barrier.

Intestinal microbial metabolites provide the substrate for the fermentation of complex dietary carbohydrates to produce short-chain fatty acids (SCFAs), as well as assist the host in harnessing maximal energy from dietary consumption (Jiang et al., 2015). The various metabolites exert varying effects on the host, from the beneficial production of signaling molecules (e.g., butyrate), to inducing mucus and other secretions, to provide the triggers that facilitate the innate mucosal system to maintain local homeostasis. In states of dysbiosis, the intestinal barrier increases in permeability as a result of a disruption to the regulation of the epithelial cell-to-cell tight junction protein network. A compromised intestinal barrier can be associated with bacterial translocation from the gut into the systemic circulation increasing the risk of sepsis. Lipopolysaccharides (LPS), a constituent of gram negative bacteria (Jiang et al., 2015), is found to be increased in the systemic circulation, indicative of dysbiosis (Boulangé et al., 2016). LPS has been associated with inducing apoptosis of lymphocytes under *in vivo* conditions (Norimatsu et al., 1995; Nielsen et al., 2012; Jiang et al., 2015) demonstrating an immune-modulatory effect. Studies have posited that a loss of lymphocytes in the intestinal mucosa is a consequence of intestinal epithelial dysbiosis and subsequent release of metabolic endotoxins (Jiang et al., 2015). LPS has also been implicated as an inductor of a pro-inflammatory environment which is conducive to MetS, IR and T2DM (Cani et al., 2008). Gram-negative bacteria containing LPS are therefore hypothesized to contribute to NAFLD development. Furthermore, dysbiosis and elevated systemic LPS can be envisaged as markers of intestinal toxicity (Nolan, 2010). Intestinal toxicity driven dysbiosis supports local mucosal inflammatory responses that is concomitant with an increase in intestinal permeability. This combined disruption of the intestinal barrier/mucosal immunity activity can promote and mediate NAFLD pathogenesis via the gut-liver axis (Littman and Pamer, 2011; Wieland et al., 2015).

## The gut-liver axis

The venous system of the portal circulation defines the gut-liver axis and highlights the close anatomical proximity and functional interactions of the gastrointestinal tract and the liver (Paolella et al., 2014; Brandl et al., 2017). The axis is described as a means of enhancing interactions between metabolites of the intestinal microbiome and receptors on the liver, which can trigger a cascade of events that culminates in IR, inflammation of the liver, and eventually the development of liver fibrosis (Paolella et al., 2014). The anatomical and functional link between the gut and liver delivers 70% of hepatic blood supply via the portal vein. The portal vein is the direct venous outflow from the intestines and thus when the intestinal mucosal barrier is compromised it exposes hepatic tissue to toxic factors derived from the intestines. Therefore, various metabolites produced by intestinal bacteria that reach the liver, have been linked to the manifestation of simple steatosis and NASH (Raman et al., 2013). Dynamic shifts in the gut-liver axis, contributed by either the physical barrier, the microbiome or the liver itself, are a result of alterations to the permeability of the intestinal epithelium and or microbial composition that have been implicated in NAFLD manifestation (Mehal, 2012). Experimental and clinical evidence increasingly implicate dysfunctions of the gut-liver axis in the development and progression of NAFLD through small intestinal bacterial overgrowth (SIBO) in conjunction with intestinal dysbiosis and increased permeability (Compare et al., 2012; Li et al., 2013; Miele et al., 2013; Vajro et al., 2013; Paolella et al., 2014). Recent experimental and clinical studies suggest that the gastrointestinal microbiome affects NAFLD pathogenesis through pathways that (i) facilitate metabolism and energy harvesting (Turnbaugh et al., 2006; Jiang et al., 2015), as described from high-fat fed mice models treated with high levels of pro-inflammatory cytokines that promote NAFLD development (Le Roy et al., 2013); (ii) dynamic interactions with the host's innate immune system where NAFLD is reported consequent to disrupted local immune cell functionality (Su et al., 2012).

## Intestinal microbial composition and NAFLD

Widespread biodiversity exists in the microbial ecosystems of humans, particularly in the intestinal tract. However, despite the extensive variety of bacteria, four main phylum dominate in the intestines: *Firmicutes, Bacteroidetes, Actinobacteria, and Proteobacteria* (Mokhtari et al., 2017), with up to 90% of microbes estimated to belong to the Firmicutes and Bacteroidetes phyla (Eckburg et al., 2005). In both human and experimental model based studies, NAFLD has been associated with altered microbiome abundance, composition and dysbiosis (Wigg et al., 2001; Mouzaki et al., 2013; Zhu et al., 2013). Analyses have also correlated changes in microbiota composition with change in disease severity (Shavakhi et al., 2013; Eslamparast et al., 2014, 2015; Rahimlou et al., 2015; Yari et al., 2016).

In reviewing the literature, studies that have analyzed the intestinal microbial composition of NAFLD patients in comparison to healthy controls have reported identifying patterns or trends that can be associated with NAFLD development (Harris et al., 2012).

Studies that have profiled the intestinal microbiome of NAFLD patients report that specific configurational and compositional shifts are associated with intestinal epithelial cell dysbiosis and the elicitation of pro-inflammatory immune responses, central to NAFLD manifestation and progression (Kirpich et al., 2015). Specific bacteria have been associated with NAFLD phenotypes, serving as both antagonists and protagonists in NAFLD pathogenesis. Studies which profiled the intestinal microbiome of healthy controls and NAFLD patients across the spectrum, were able to identify families, genera and phyla that differed significantly in their abundance between the healthy controls and those with a NAFLD diagnosis. Whilst data is inconsistent regarding the association between the intestinal microbiome profile and NAFLD, patterns are emerging which highlight potential relationships between bacterial types and host health (Lau et al., 2015). A study comparing subjects diagnosed with NAFLD compared to lean adults, reported that gram negative bacteria were significantly enriched (*P* = 0.009) and gram positive bacteria were markedly decreased (*P* = 0.001) in the NAFLD cohort (Wang et al., 2016). Findings from similar studies confirm the strong relationship between the composition and configuration of the intestinal microbiome and fatty liver histologies, suggesting that adverse shifts in intestinal microbiome profiles are related to the development of NAFLD (Wang et al., 2016). In a prospective cross sectional study, a 20% increase in the *Bacteroidetes* phylum (*p* = 0.005) and a 24% decrease in *Firmicutes* (*p* = 0.002) was found in healthy controls in comparison to NAFLD patients (Wang et al., 2016). Interestingly, among the species belonging to the *Firmicutes* phylum, SCFA-producing bacteria were significantly decreased. Specific microbiome signatures of intestinal bacteria that are reported to be associated with significant reductions in butyric acid (Consolandi et al., 2015) may comprise a significant marker for the depletion of intestinal bacterial species that are important for the maintenance of intestinal barrier integrity and innate mucosal immunity equilibrium. However, currently it is difficult to identify which microbiome differences are causal and which are coincidental in the development of intestinal barrier dysbiosis and moreover NAFLD. Variations between studies, which profile microbiome composition of NAFLD patients, may in part be accounted for by different analytical techniques that have been employed. Furthermore, differences in study design, including anthropometric measures, markers used for NAFLD diagnosis as well as ultra-sonographic vs. biopsy NAFLD diagnosis adds additional discrepancies. As such Mouzaki et al. (2013), reported an inverse relationship between NASH diagnosis and the proportion of the phyla *Bacteroidetes* detected. These results were contrasted by Zhu et al (Zhu et al., 2013) who reported that NASH diagnosis was accompanied by higher levels of alcohol-producing bacteria and endogenous levels of ethanol, a result that was supported by Wong et al. (2013).

The intestinal microbial variations were also observed at the genus level, with data purporting to *Ruminococcus* and *Roseburia* genera being shown to be inconclusive. Whilst Zhu et al. (2013) and Raman et al. (2013) reported a non-significant decrease in the abundance of *Ruminococcus* in NAFLD patients compared to a group of healthy controls, whereas Del Chierico et al. (2017) and Jiang et al. (2015) found an increase in the genus' abundance in NAFLD patients.

Despite the various limitations, preliminary studies highlight and support the hypothesis that configurational shifts in the intestinal microbiome composition may contribute to the development and progression of NAFLD. Whilst further studies on a larger scale with accurate measures and controlled variables are warranted, the potential contribution of intestinal microorganisms and their metabolites are implicated in the pathophysiology of NAFLD. Studies that administer probiotics/prebiotics, which posit to encourage the intestinal microbiome to re-establish intestinal-mucosal macrophage crosstalk homeostasis that then translates to reducing the progression of NALFD are very much warranted.

## Mechanisms linking the intestinal microbiota and NAFLD

Research identifies several mechanisms by which the intestinal microbiome cohort-arrangement can affect NAFLD pathogenesis and maintenance. Increased intestinal permeability (Jandhyala et al., 2015), small intestine bacterial overgrowth (SIBO) (Zhu et al., 2013) and elevated serum endotoxin like lipopolysaccharide (LPS) (Brun et al., 2007; Soares et al., 2010), have been reported by studies with NAFLD patients, with varying disease severity and staging (Elshaghabee et al., 2016). LPS, a component of gram-negative bacteria, is elevated in cases of bacterial overgrowth and increased intestinal permeability, yielding hepatoxic effects through the activation of TLR4 and initiation of a cascade of pro-inflammatory innate immune responses (Wigg et al., 2001; Soares et al., 2010).

Experimental and clinical data suggests that SIBO and a disturbed intestinal epithelial barrier are involved in NAFLD pathogenesis (Bode et al., 1987; Purohit et al., 2008; Wan et al., 2016). Furthermore, investigations have shown that the serum from NAFLD patients have elevated levels of LPS-binding protein, TLR-4, and TNF-α in hepatic tissue (Ruiz et al., 2007; Wan et al., 2016).

The gut microbiota also contributes to NAFLD pathogenesis through enriched numbers of ethanol-producing bacteria, primarily *Escherichia coli* (Small et al., 2013; Zhu et al., 2013; Jiang et al., 2015). The alcohol produced by these bacteria are reported to be involved in compromising intestinal barrier integrity which instigates inflammatory activity and ultimately hepatoxic events. Ethanol is a common and dominant metabolite of numerous resident intestinal microbes. As a product of hetero-lactic organisms, endogenously produced ethanol is implicated as a pro-inflammatory hepatoxic factor in NAFLD pathogenesis (Cope et al., 2000; Baker et al., 2010). Linked with increased concentrations of serum ethanol, enrichment in the presence of alcohol-producing intestinal bacteria, like *E. coli*, has been demonstrated to increase intestinal epithelial permeability (Aron-Wisnewsky et al., 2013; Jiang et al., 2015).

**Figure 2:**
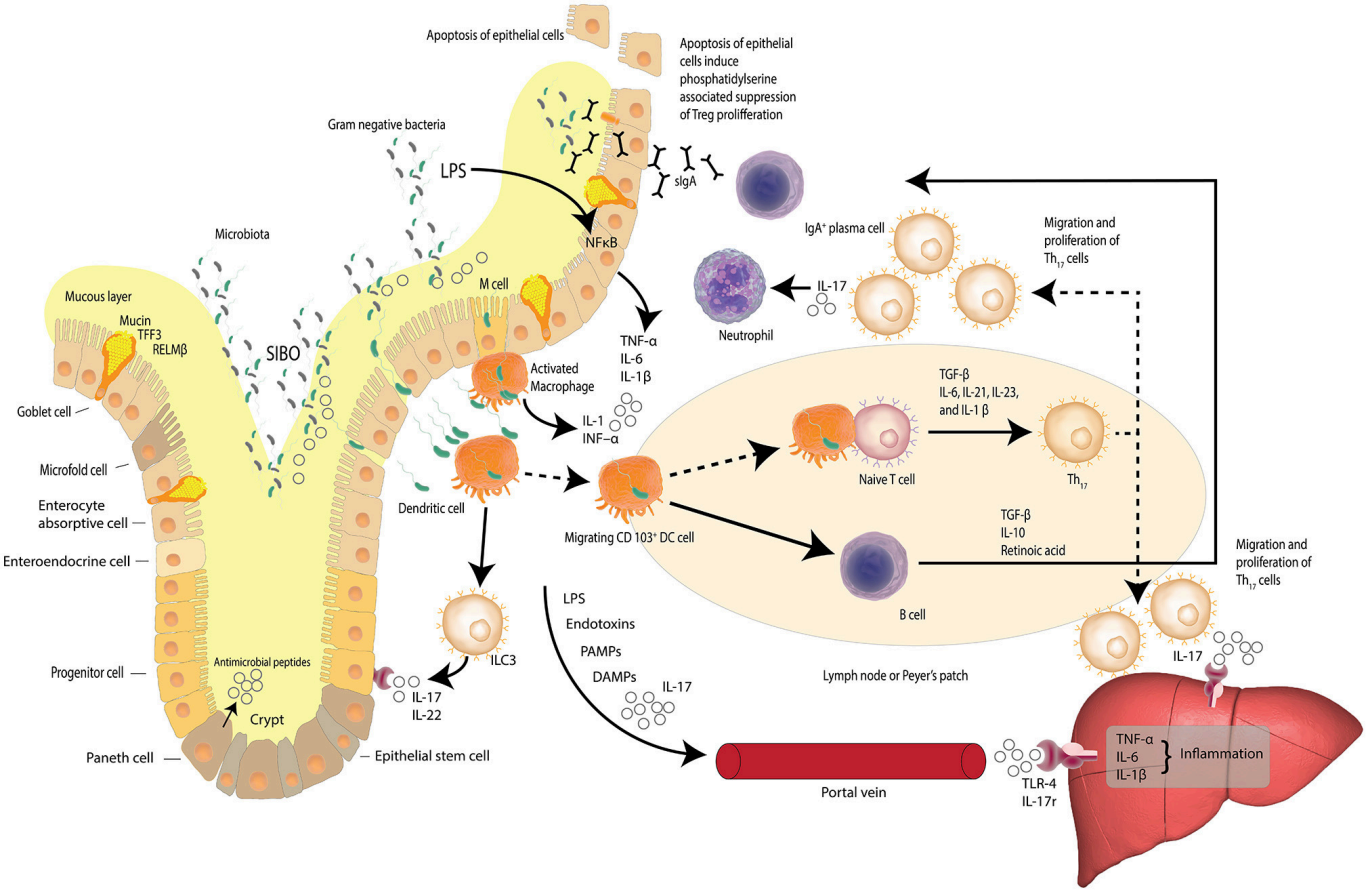
Dysbiosis and the gut-liver axis. Increased intestinal permeability, small intestine bacterial overgrowth (SIBO) and elevated serum endotoxin like lipopolysaccharide (LPS) is found in patients with NAFLD. LPS, a hepatoxic component of gram-negative bacteria, is elevated in cases of SIBO. Increased intestinal permeability leads to increased bacterial translocation. Activated macrophages release inflammatory cytokines and dendritic cells stimulate the differentiation of naïve T cells to pro-inflammatory Th17 cells induced by TGF-β, IL-6, IL-23, and IL-1β. Th17 cells secrete neutrophil-activating IL-17. Lymphoid Th17 also migrate to the liver where IL-17 stimulates monocytes, Kupffer cells, biliary epithelial cells, and stellate cells, to secrete pro-inflammatory cytokines and chemokines—inducing liver inflammation. LPS, bacterial endotoxins, IL-17, pathogen-associated molecular patterns (PAMPs) and damage-associated molecular patterns (DAMPs) may reach the liver via the portal vein and induce inflammation via Toll-like receptor 4 (TLR-4) and interleukin-17 (IL-17) receptors. The gut-liver axis is underlined by the fact that at least 70% of the liver's blood supply is delivered directly from the intestinal tract via the portal vein outflow of the intestine. (Zheng, 2013; Carding et al., 2015; Robinson et al., 2015; Fukui, 2016; Kumar et al., 2016) Adapted from Peterson and Artis (2014).

A prelude to the liver's averseness to excessive accumulation of FFAs, is intestinal dysbiosis. Dysbiosis is the concept that describes compositional alterations away from the conventional symbiotic intestinal microbiota that may be associated with pathology within the host and is visually described in Figure 2. Herein we further posit that compositional alterations in the intestinal microbiota adversely affect the intestinal epithelial barrier exacerbating *epithelial cell dysbiosis*. It has been shown that intestinal epithelial cell disruption can directly adversely affect intestinal resident macrophages and act as critical effector cells in the initiation of inflammation in the pathogenesis of metabolic diseases (Chawla et al., 2011). Disturbances of the TJP network which link the epithelial cells to form the intestinal barrier is a central regulatory mechanism, facilitating selective permeability across the intestinal mucosa and limiting bacterial translocation. Examining the duodenum of NAFLD patients and healthy adults has revealed that in comparison to NAFLD patients, (Jiang et al., 2015) the TJP network was significantly more intact in the duodenum of a healthy adult, with regular alignment and extensive abundance of the microvilli(Jiang et al., 2015). These observations are in stark contrast to the significantly wider gaps and disrupted TJPs reported in NAFLD patients, suggesting a loss of barrier integrity with a consequent increase in bacterial translocation through increased intestinal permeability (Briskey et al., 2016). Additional assessment of the serum biomarkers of the TJPs, including occludin proteins, which are structural components of the tight junctions, have been reported with significantly higher levels in the intestinal mucosa of healthy adults compared to NAFLD patients (Jiang et al., 2015). Measuring the serum levels of the proteins that comprise the structural backbone of the intestinal TJP network lends further supportive data to the hypothesis that intestinal mucosal permeability is greater in NAFLD patients than lean subjects or controls, suggesting intestinal epithelial dysbiosis is a causal factor in NAFLD pathogenesis.

With the establishment of the intestinal microbiota as a participant in the onset and maintenance of low-grade systemic inflammation, the probing of the intestinal microbiome as a potential therapeutic target for extra-intestinal inflammatory conditions, such as NAFLD begun.

The supposition that the intestinal microbiome could indirectly and adversely influence the physiological function of an end–organ such as the liver, by contributing pro–inflammatory activity in the intestinal mucosa, is a novel concept with biological plausibility. As an example, the uptake of Shiga-toxin from the pathogen enterohemorrhagic *E. coli* by M cells and the underlying macrophages in the Peyer's patches is a critical step that teaches about bacterial translocation. This step has been correlated to the efficiency of the infection by the pathogen (Etienne-Mesmin et al., 2011). Numerous bacterial pathogens and their products cross the epithelial barrier though M cell junctions that are then subsequently captured by intestinal resident macrophages (Alisi et al., 2017). Moreover, the LPS components of bacteria are ligands of TLR4 that are expressed on various immune cells, including intestinal macrophages (Vijayan et al., 2017). TLR4 in the M1 configuration is a mediator of inflammation that may imply that increased LPS/TLR4 signaling could be a driving factor in the accelerated inflammation process in patients with NAFLD (Alisi et al., 2017). LPS also induces activation of genes on macrophages such as the early growth response gene 1 (EGR1) as well as the LPS-induced expression of TNF-α, an action that is directly mediated through EGR1 and NF-kB (Xu et al., 2001). Therefore bacterial products that activate macrophages and other immune cells to produce pro-inflammatory mediators can trigger inflammation in an end organ such as the liver (Tateya et al., 2013). Signals released in response to microbial dangers are absorbed on a backgorund of increased and dysregulated intestinal barrier permeability. These signals are recognized by PRRs, including TLR-4 (Szabo et al., 2010; Wan et al., 2016), and when sensed by NLRP3, support the hypothesis that inflammasome-driven microflora are potential drivers of NAFLD onset (Wan et al., 2016).

Intestinal homeostasis is pivotal in optimal functionality of the innate immune system and hinges on macrophages eliminating pathogenic bacteria and their particles (Vitetta, 2016). Activated macrophages play a dual role within the innate immune system (Sansonetti, 2002; Vitetta, 2016). Firstly, they help to elicit appropriate immune responses to detected microbial proteins by facilitating the presentation of antigens to T lymphocytes (Sansonetti, 2002; Vitetta, 2016). Secondly, activated macrophages serve as a secretory source for an array of cytokines that regulate the activation of T cell lymphocytes, including IL-1, INF–α, and cytotoxic proteins (Sansonetti, 2002; Vitetta, 2016). The overall action of the macrophage within the immune responses rely on their ability to neutralize exogenous antigens, cellular debris, insoluble particles, and activated clotting factors via phagocytic activity (Tacke, 2017).

## Reprise

NAFLD's pathway to pathogenicity is characterized by the presence of ectopic fat within hepatocytes that results from an imbalance in the levels of lipogenesis and lipolysis (Machado and Cortez-Pinto, 2014). Triglycerides are synthesized from FFAs that are reported to accumulate in the liver. Therefore, it is envisaged that the concentration of FFAs function as a regulator of lipogenesis in the liver.

Previous studies have associated bacterial phyla, families, or even single genera with obese or lean phenotypes, with an increased lactobacilli count and decreased *Bacteroidetes* presence associated with leanness (Armougom et al., 2009). In support of this, an increased abundance of genus' of the *Bifidobacterium animalis* or *Lactobacillus* species were associated with weight management and a healthy body weight (Million et al., 2012). *Lactobacillus reuteri* has specifically been identified as being associated with an obese phenotype (Million et al., 2012). Assessing intestinal microorganisms for possible correlations with NAFLD is a biologically plausible next step in progressing an understanding of how much influence the intestinal microbiome as a metabolic and immunological organ may have on the development and progression of NAFLD.

Methodological and technological difficulties have largely prevented robust and definitive data from studies that specifically assess the intestinal microbiota of adults with NAFLD and the health of the intestinal mucosal barrier as well as a need for further knowledge into what constitutes a healthy microbiota. When the mucosal barrier of the intestine, which also serves as the largest immune area of the intestinal immune system, is impaired and disrupted, the liver is exposed to intestinal-derived bacterial factors, which are potentially hepatoxic through the gut-liver axis.

A majority of the current literature details the involvement of the innate branch of the immune system in pro-inflammatory pathways leading to NAFLD pathogenesis and progression. However, the adaptive immune system is also implicated in NAFLD development (Ganz and Szabo, 2013; Sutti et al., 2016). Linking the innate and adaptive branches, natural killer (NK) cells are abundant in hepatic tissue and have been reported to influence the development of liver injury and fibrotic deposition that spark NASH materialization (Ganz and Szabo, 2013). Studies in both animal and human models have found a decrease in circulating NK cells in obese subjects compared to lean counterparts (Ganz and Szabo, 2013), with further exploratory investigations suggesting a reduction in their levels and thus activity may in turn increase the sensitivity of obese patients to develop progressive forms of NAFLD, including cirrhosis (Radaeva et al., 2006). A subset of NK cells, the natural killer T (NKT) cells, also known for their exhibition of both innate and adaptive immunity features, serve to regulate hepatic immune responses by secreting both Th1 and Th2 cytokines (Godfrey et al., 2000). Experimental and clinical data indicates that a depletion in NKT cells can lead to the chronic pro-inflammatory environment that can accompanies hepatic steatosis (Li et al., 2005; Ronchi and Falcone, 2008).

With no effective medication having yet been tested for managing or treating NAFLD and the only universally accepted treatment strategy being lifestyle modifications that focus of weight loss, novel therapeutic agents are being pursued in an endeavor to address the rise of NAFLD as one of the most common non-communicable liver disease world-wide (Volynets et al., 2012).

Whilst lifestyle modification recommendations encourage weight loss, this approach requires significant commitment and efficiency decreases over the long term due to waning dedication. Intestinal microbial manipulation through the administration of probiotics presents as an attractive therapeutic adjunct. In an attempt to reduce intestinal epithelial inflammation, probiotics shift the intestinal microbial community toward beneficial bacterial communities such as the *Parabacteroides, Prevotella*, and *Oscillibacter* (Ohland and MacNaughton, 2010). These microbial communities are well known to produce anti-inflammatory metabolites such as SCFAs (Schwiertz et al., 2010). SCFAs, such as butyrate, serve as important facilitators in the harvesting of energy and harnessing it for peripheral tissues and the intestinal epithelia (Elshaghabee et al., 2016). *Oscillibacter* and *Parabacteroides* are associated with T-cell differentiation by enhancing and maintaining the IL-10 producing Treg cells (Arpaia et al., 2013). Moreover, a probiotic formulation that attenuated hepatocellular carcinoma in a murine model offered further insight on how the intestinal microbiota influences the regulation of T-cell differentiation of mucosal immunity in the intestines and in turn, there is down-regulation of the level of pro-inflammatory cytokines (Li et al., 2016). A recently completed murine study by our group reported attenuation of steatosis by 60% in a high fat diet model of NAFLD (Briskey et al., 2016). As such the administration of probiotics to attenuate the progression of NAFLD is both clinically plausible and very much warranted.

Despite increasing focus and attention directed at identifying the mechanisms by which NAFLD develops and progresses, ambiguity remains surrounding the driving factors and molecular pathways that result in NAFLD onset. Immunological mechanisms, including the collaboration of the consequences of innate immunity, adaptive immunity, and TLR receptor signaling dysfunction with the gut-liver axis are each posited to contribute to disease pathogenesis and maintenance.

This narrative review has highlighted the requisite for the completion of a systematic literature review detailing the association between innate immune responses triggered by intestinal epithelial inflammation and dysbiosis and the onset of extra-intestinal pathologies, such as NAFLD. The intestinal microbiome is a significant environmental factor in NAFLD pathogenesis, specifically through effects on intestinal barrier integrity. A review of the literature which explores the correlation between changes in intestinal integrity, intestinal permeability, and therefore endotoxin translocation will help elucidate the specific patterns or profiles of intestinal microorganisms that are of interest in NAFLD manifestation and therefore relevant for therapeutic target purposes. Furthermore, such a review may also define bacterial species that elicit a hepatoprotective effect on the microbiome and extra-intestinal inflammation.

## Author contributions

ES and LV: Conception and design of commentary, review; ES, LV, MT, and TP: Read, amended and approved final version of manuscript.

### Conflict of interest statement

The authors declare that the research was conducted in the absence of any commercial or financial relationships that could be construed as a potential conflict of interest
